# Long noncoding RNA LINC01111 suppresses pancreatic cancer aggressiveness by regulating DUSP1 expression via microRNA-3924

**DOI:** 10.1038/s41419-019-2123-y

**Published:** 2019-11-25

**Authors:** Shutao Pan, Ming Shen, Min Zhou, Xiuhui Shi, Ruizhi He, Taoyuan Yin, Min Wang, Xingjun Guo, Renyi Qin

**Affiliations:** Department of Biliary-Pancreatic Surgery, Affiliated Tongji Hospital, Tongji Medical College, Huazhong University of Science and Technology, 1095 Jiefang Avenue, 430030 Wuhan, Hubei China

**Keywords:** Pancreatic cancer, Long non-coding RNAs

## Abstract

Dysfunction in long noncoding RNAs (lncRNAs) is reported to participate in the initiation and progression of human cancer; however, the biological functions and molecular mechanisms through which lncRNAs affect pancreatic cancer (PC) are largely unknown. Here, we report a novel lncRNA, LINC01111, that is clearly downregulated in PC tissues and plasma of PC patients and acts as a tumor suppressor. We found that the LINC01111 level was negatively correlated with the TNM stage but positively correlated with the survival of PC patients. The overexpression of LINC01111 significantly inhibited cell proliferation, the cell cycle, and cell invasion and migration in vitro, as well as tumorigenesis and metastasis in vivo. Conversely, the knockdown of LINC01111 enhanced cell proliferation, the cell cycle, and cell invasion and migration in vitro, as well as tumorigenesis and metastasis in vivo. Furthermore, we found that high expression levels of LINC01111 upregulated DUSP1 levels by sequestering miR-3924, resulting in the blockage of SAPK phosphorylation and the inactivation of the SAPK/JNK signaling pathway in PC cells and thus inhibiting PC aggressiveness. Overall, these data reveal that LINC01111 is a potential diagnostic biomarker for PC patients, and the newly identified LINC01111/miR-3924/DUSP1 axis can modulate PC initiation and development.

## Introduction

Pancreatic cancer (PC) is reported to be one of the most malignant tumors in adults and ranks fourth in terms of cancer-related deaths for both men and women^1^. Despite continuous improvements in medical technology over the past decades, including surgical resection and adjuvant medical therapy, the 5-year survival rate of PC patients remains approximately 7%^2,3^. Moreover, PC patients are often diagnosed at an advanced stage of cancer because of the atypical symptoms of PC and the limitations of diagnostic techniques^4^. Thus, in order to improve the prognosis of PC patients, the identification of novel diagnostic biomarkers and the development of innovative molecular therapeutic strategies are now urgently needed.

Long noncoding RNAs (lncRNAs) are defined as noncoding RNAs more than 200 bp in length with no or limited protein-encoding potential that are involved in various biological processes, including cell differentiation, metastasis, immune responses, autophagy, and apoptosis^5,6^. Recently, an increasing number of researches have been reported to research on the important roles of lncRNAs in cancer carcinogenesis and development^7–10^. These data suggest that lncRNAs may play an increasingly important role in the early diagnosis and molecular targeted therapy of human cancer.

MicroRNAs (miRNAs) are noncoding RNAs with length of approximately 23 nt that exert their gene-regulatory roles in cells by targeting the paired mRNAs of protein-encoding genes, leading to RNA degradation or post-transcriptional repression^11^. As reported, miRNAs are involved in various activated oncogenic pathways and function as regulators of the initiation and progression of human cancer^12^. Recently, studies have revealed that a number of lncRNAs act as competitive endogenous RNAs (ceRNA) through their miRNA pairing sites, by which lncRNAs can restore the post-transcriptional repression of mRNAs via competing with miRNAs^10^^,11,13^^,14^.

In the present study, we aim to identify lncRNAs that may have an impact on PC initiation and progression. We found a long intergenic noncoding RNA, LINC01111, that was clearly downregulated in PC tissues and plasma of PC patients. LINC01111 exerts its tumor suppressive effects via the inactivation of the stress-activated protein kinase/c-Jun NH2-terminal kinase (SAPK/JNK) signaling pathway by upregulating a related gene, dual specificity phosphatase 1 (DUSP1), which inhibits SAPK/JNK phosphorylation^15^. Furthermore, our study reveal that LINC01111 upregulates DUSP1 expression by sequestering miR-3924. Overall, LINC01111 is highly associated with PC malignancies and may offer a promising treatment approach for PC patients.

## Materials and methods

### Patients and samples

We collected tissues and plasma of newly diagnosed and surgically treated patients in Tongji Hospital of Huazhong University of Science and Technology during the period from May 2016 to Aug. 2018 (see detail information in supplementary files). All patients participating in this study received informed consent. We performed histological grading according to the seventh TNM staging of the International Union against Cancer (UICC)/American Joint Committee on Cancer (AJCC) system. Fresh tissue and plasma samples were immediately frozen in liquid nitrogen and preserved at −80 °C until RNA separation. The Human Ethics Committee of Tongji Hospital of Huazhong University of Science and Technology approved this study, and all the studies were in line with the principles of the Declaration of Helsinki.

### Selection of candidate lncRNAs

We screened in the Gene Expression Omnibus (GEO) database for lncRNAs that were differentially expressed in PC. On the one hand, there are 6 human microarray datasets (GSE61166 [Chen et al., 2014], GSE89139 [Zhou et al., 2016], GSE86436 [Liu et al., 2016], GSE100232 [Yu et al., 2017], GSE101094 [Liang et al., 2017] and GSE57144 [Li et al., 2017]) introducing the expression data from 46 PC tissues and 40 normal pancreatic tissues. On the other hand, there is one human microarray dataset (GSE71008 [Yuan et al., 2016]) introducing the expression data from plasma of 6 PC patients and 6 healthy subjects. The microarray probes were reannotated to lncRNAs. The levels of differentially expressed lncRNAs were calculated by using R package limma.

### Cell culture

The human normal pancreatic duct epithelial (HPDE) cell line and human PC cell lines (PANC-1, MIA PaCa-2, SW1990, Capan-2, Panc 03.27, BxPC-3, CFPAC-1) were purchased from American Type Culture Collection (ATCC, Manassas, VA, USA). HPDE, Panc 03.27, BxPC-3, and CFPAC-1cell lines were cultured in RPMI 1640 medium (Gibco, NY, USA) supplemented with 10% fetal bovine serum (FBS, Gibco). PANC-1, MIA PaCa-2, SW1990, and Capan-2 cell lines were cultured in Dulbecco’s Modified Eagle’s Medium (DMEM, Gibco) supplemented with 10% FBS (Gibco). All cell lines were authenticated and tested to be mycoplasma-free. All cell lines were cultured in a humidified incubator containing 5% CO_2_ at 37 °C.

### RNA isolation and quantitative real-time PCR analysis

Total RNA was isolated from PC cells and tissues using the TRIzol reagent (Takara, Dalian, China) in accordance with the manufacturer’s introductions. Plasma total RNA was extracted using a miRNeasy Micro Kit (QIAGEN, Duesseldorf, Germany) in accordance with the manufacturer’s introductions. The quality of RNA samples were evaluated before quantitative real-time PCR (qRT-PCR). One μg of total RNA was used for synthesis of the first-strand cDNA using the reverse transcriptase cDNA synthesis kit (Takara). The obtained cDNA was then analyzed by qRT-PCR analysis using the SYBR Green PCR kit (Takara) and the 7500 Fast real-time PCR system (AB Applied Biosystems) in accordance with the manufacturer’s introductions. Human GAPDH and U6 genes were used as internal controls. The 2^−ΔΔCt^ method was used to calculate the relative expression of genes. See the primer sequences of genes in the supplementary file.

### Cell proliferation assay

The CCK-8 kit (Dojindo Laboratories Co. Ltd., Kumamoto, Japan) was used to assess cell viability in accordance with the manufacturer’s introductions. In detail, we seeded cells (3 × 10^3^ per well) into the 96-well plates with each well containing 200 μl of culture medium supplemented with 10% FBS. We have six replicates for each sample. At the appointed time point, solution with 100 μl of fresh medium and 10 μl of CCK-8 solution was added into each well. After incubated for 1–2 h at 37 °C, the absorbance was recorded at 450 nm using the Quant ELISA Reader (BioTek Instruments, USA). Survival rate % = (OD treatment−OD blank)/(OD control−OD blank) × 100%.

### Colony formation assay

We seeded cells (1000 per dish) into 6-cm-sized dishes and cultured them for 2–3 weeks. The cells were then fixed with 4% paraformaldehyde for 30 min and subsequently stained with 1% crystal violet for 30 min. To assess the cell viability, we counted and calculated the colonies with diameters greater than 100 μm.

### EdU incorporation assay

We seeded cells into the 6-well plates and cultured them until they reached an appropriate density. We then added the 5-ethyl-2′-deoxyuridine (EdU) to the culture medium and performed the immunofluorescence staining using the EdU kit (RiboBio, Guangzhou, China) in accordance with the manufacturer’s introductions. EdU-positive proliferating cells are expressed as a percentage of the control level.

### Scratch wound healing assays

We seeded the cells into the 6-well plates and cultured them until they fully fused. We then manually scratched the cell monolayer using a 200-μl pipette tip and washed out the floating cells with phosphate-buffered saline (PBS). After that, cells were cultured for 48 h in culture medium supplemented with 1% FBS. The phase contrast microscope (Niko Corporation) was used to capture the images and the Image Pro Plus v6.0 software package (Media Cybernetics Inc., Bethesda, MD, USA) was used to measure the migration areas of cells.

### Matrigel invasion assay

The transwell assay was performed using the 24-well transwells (8 μm pore size, corning, NY, USA) precoated with or without the Matrigel (BD Bioscience, San Jose, CA, USA) in accordance with the manufacturer’s introductions. In brief, We seeded cells (3 × 10^4^ per chamber) in 200 μl of serum-free medium into the upper chamber (with or without Matrigel) and added 800 μl of culture medium supplemented with 10% FBS to the bottom well as a chemoattractant. After that, the cells were cultured for 30–36 h, then fixed with 4% paraformaldehyde for 30 min and subsequently stained with 1% crystal violet for 30 min. Images of the bottom surface of the chamber were captured using the phase contrast microscope (Niko Corporation) and cells migrated to the bottom surface were quantified.

### Western blotting and antibodies

The total cellular protein was isolated using the RIPA buffer (Boster Biological Technology, Wuhan, China). Cytoplasmic and nuclear protein fractionation and isolation were performed using the Protein Extraction kit (Boster Biological Technology) in accordance with the manufacturer’s introductions. The SDS-PAGE Electrophoresis System was used to extract protein. Target protein was then transferred from the SDS-PAGE gel to the PVDF membrane (Millipore, MA, USA), incubated overnight at 4 °C with primary antibody, incubated for 2 h at RT with a specific secondary antibody conjugated to horseradish peroxidase and finally examined with the ChemiDoc XRS System (Bio-Rad Laboratories, USA). The following antibodies were purchased: anti-SAPK/JNK (#9252), anti-Phospho-SAPK/JNK (Thr183/Tyr185) (#4688), anti-c-Jun (#9165), anti-Phospho-c-Jun (Ser73) (#3270), anti-Cyclin D1 (#2978), anti-Cyclin A2 (#4656) antibody were obtained from Cell Signaling Technology (Beverly, MA, USA); anti-DUSP1 (A2919), anti-DUSP4 (A2726), anti-DUSP10 (A8748), anti-DUSP16 (A10155), anti-MAP2K4 (A14781), anti-Phospho-MAP2K4-S257/T261 (AP0541), anti-MAP2K7 (A2186) antibody were obtained from Abclonal Biotechnology (Wuhan, China); anti-GAPDH (60004-1-Ig) was obtained from Proteintech Group (Chicago, IL, USA); anti-PCNA (sc-25280), anti-Ki-67 (sc-15402), anti-N-cadherin (sc-53488), anti-Vimentin (sc-80975) antibody were obtained from Santa Cruz Biotechnology (Santa Cruz, CA, USA); SAPK/JNK signaling pathway inhibitor SP600125 (HY-12041) were obtained from MCE (Monmouth, NJ, USA).

### Immunofluorescence

After fixed with 4% paraformaldehyde for 30 min and permeabilized with 0.2% TritonX-100 (Boster Biological Technology) for 5 min, the cells were blocked in 5% bovine serum albumin (BSA, diluted in PBS) for 30 min. The cells were then incubated overnight at 4 °C with primary antibodies. Primary rabbit polyclonal antibody Phospho-SAPK/JNK (Thr183/Tyr185) (#9255, CST, MA, USA) was diluted 1:50 in blocking buffer. Fluorescein isothiocyanate (FITC)-conjugated secondary antibody (Proteintech, Chicago, USA) was diluted 1:200 in blocking buffer and applied to cells at RT in the dark for 1 h, followed by stained with 4′, 6-diamidino-2-phenylindole (DAPI, 1:1000) at RT in the dark for 10 min. The confocal fluorescence microscopy (PerkinElmer, Waltham, Massachusetts, USA) was used to capture the immunofluorescence images.

### RNA microarrays

The Agilent RNA 6000 Nano kit (Waltham, MA, USA) was used to extract total RNA from LV-LINC01111 and NControl cell samples in accordance with the manufacturer’s introductions. Then total RNA was subjected to microarray analysis using the Prime View Human Gene Chip (Affymetrix, Waltham, MA, USA). After that, the Gene Chip Hybridization Wash and Stain kit (Affymetrix) was used to perform the RNA labeling and hybridization in accordance with the manufacturer’s introductions. Data were deposited and the accession code is GSE138420.

### RNA fluorescence in situ hybridization and in situ hybridization

The Tyramide Signal Amplification System (PerkinElmer, USA) was used to measure the LNA probe signals for the FISH experiments. In brief, after incubation with horseradish peroxidase (HRP)-conjugated anti-DIG, the signal was detected. The signal was then amplified with tetramethylrhodamine (TRITC)-conjugated tyramine. The fluorescence microscopy (PerkinElmer) was used to capture the images. To perform the RNA ISH experiment, the RNA ISH kit (BersinBi, Beijing, China) was used in accordance with the manufacturer’s introductions. In brief, after fixed with 4% paraformaldehyde for 20 min and washed using distilled water, the tissue slides were treated with 1% pepsin (diluted in 10 mM HCl), followed by an incubation with 20 nM ISH probe diluted in the hybridization buffer (100 mg/ml dextran sulfate, 10% formamide in 2x SSC) for 3 min at 90 °C. Hybridization was performed for 18 h at 37 °C, and a washing step followed. After that, the tissue slides were incubated with digoxin antibody for 1 h. For detection of signals, BAD was applied to the samples. The Aperio ImageScope System was used to capture the ISH images. Each sample was separately reviewed and scored by two pathologists who were blinded to the clinical outcomes. To calculate the relative expression quantity, we need the product of staining intensity and positive rate of cells for each sample.

### Luciferase reporter assay

To construct the human DUSP1 3′UTR luciferase reporter (DUSP1-WT) and the miR-3924 target site-mutation DUSP1 3′UTR luciferase reporter (DUSP1-mut), we have the full-length 3′-UTR of DUSP1 mRNA and the mutant derivative devoid of the miR-3924 target site amplified and cloned into the XbaI site of psi-CHECK2 luciferase reporter vector (Promega, WI, USA). Similarly, Wild-type LINC01111 (LINC01111-WT) and the miR-3924 target site-mutation LINC01111 (LINC01111-mut) were cloned into the XbaI site of the vector. The DNA sequencing technique was used to confirm nucleotide sequences of the plasmids constructed. Lipofectamine 2000 (Invitrogen, CA, USA) was used when co-transfecting plasmids and mimics-miR-3924 or mimics-NControl (RiboBio, Guangzhou, China) into Human Embryonic Kidney (HEK) 293 T cells. After 48 h, the Dual-Luciferase Assay system (Promega) was used to assay the luciferase activities in accordance with the manufacturer’s instructions.

### Animal experiments

For the tumorigenicity assays, 4-week-old female Balb/c-nude mice (from Beijing Vital River Laboratory Animal Technology Co., Ltd., Beijing, China) were divided randomly into 4 groups (10 per group) and 2 × 10^6^ transfected PC cells were injected subcutaneously into the upper right flank of each mouse. The diameter of each xenograft tumor was measured using a Vernier caliper every 3 days. For the liver metastasis assays, 4-week-old female Balb/c-nude mice were divided randomly into 4 groups (6 per group) and 2 × 10^6^ transfected PC cells were injected into the spleen of each mouse. About 4–6 weeks after inoculation, mice were euthanized for tumor tissues and organs. The harvested samples were immediately weighed, imaged, fixed with 4% paraformaldehyde, embedded in paraffin, and subjected to HE staining and IHC staining. No blinding was done. All mice were housed under specific pathogen-free conditions and all surgeries were conducted under anesthesia with sodium pentobarbital. All animal experiments were approved by the Committee on Ethics of Animal Experiments of Tongji Medical College, Huazhong University of Science and Technology. All experimental procedures and animal welfare were conducted according to the ARRIVE (Animal Research: Reporting In Vivo Experiments) guidelines.

### Statistical analyses

For statistical significance, data were analyzed using GraphPad Prism 6.0 software (GraphPad Software Inc., CA, USA) and SPSS 22.0 software (SPSS Inc., IL, USA). The results are presented as mean ± standard deviation (SD). The quantitative data were analyzed by a two-tailed Student’s *t*-test. The categorical variables were analyzed by a *χ*^2^-test. Survival analysis was conducted using the Kaplan–Meier method and analyzed by the log-rank test. *P*-value < 0.05 was considered to be statistically significant.

## Results

### Loss of LINC01111 in PC significantly correlates to poor prognosis of PC patients

**Fig. 1:**
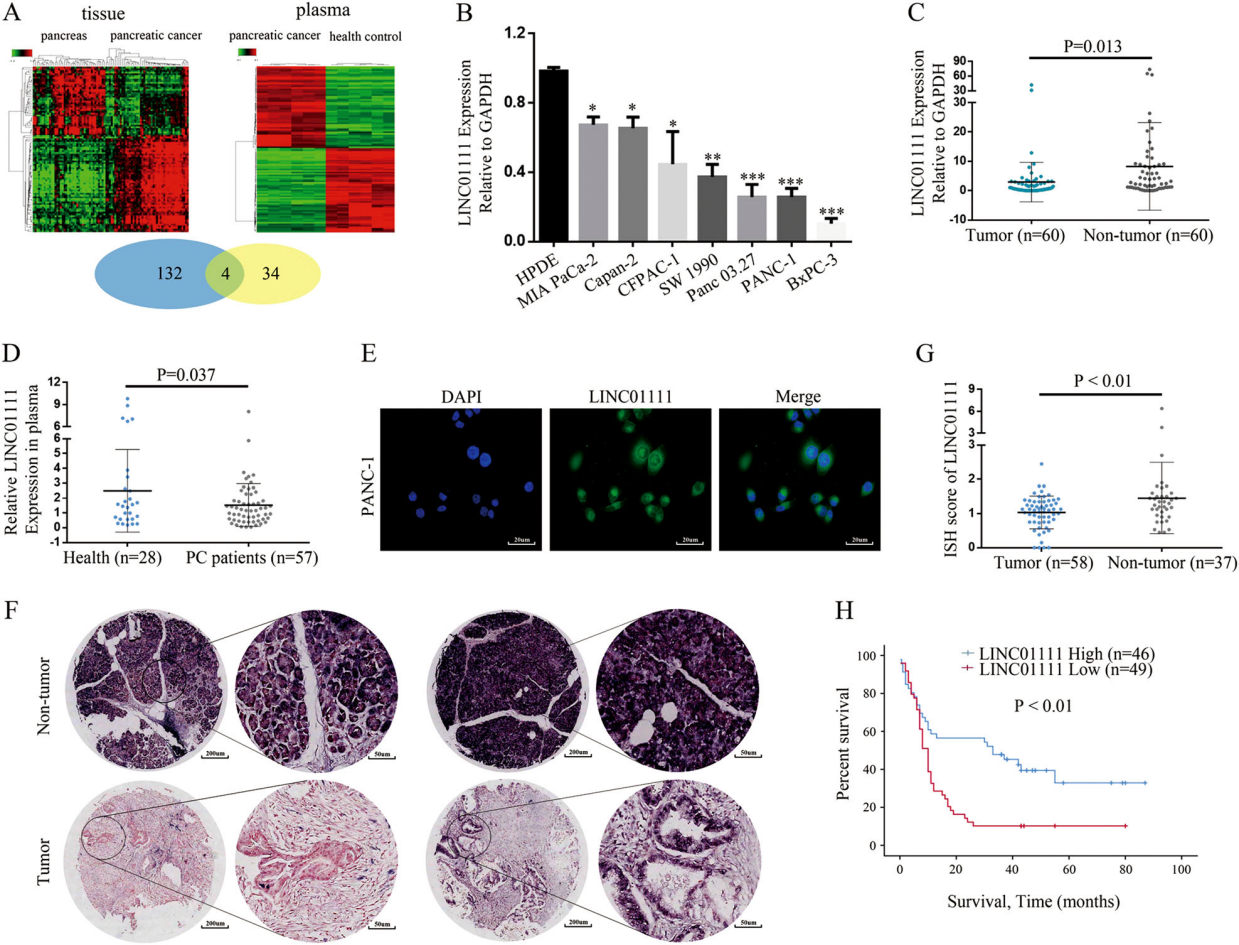
LncRNA candidate LINC01111 is clinically relevant in PC. **a** Cluster analysis of lncRNAs in PC tissues vs. normal pancreatic tissues and lncRNAs in plasma from PC patients vs. health control people. **b** PCR analysis of LINC01111 expression in PC cell lines, *p < 0.05, **p < 0.01, ***p < 0.001. **c** PCR analysis of LINC01111 expression in PC tissues (*n* = 60) vs. adjacent normal tissues (*n* = 60). **d** PCR analysis of LINC01111 expression in plasma of PC patients (*n* = 57) vs. health control people (*n* = 28). **e** Representative images of FISH detecting expression of endogenous LINC01111 in PC cells. **f** Representative images of ISH detecting expression of LINC01111 in paraffin-embedded PC specimens and adjacent normal tissues. **g** ISH score of LINC01111 expression in PC tissues (*n* = 58) vs. adjacent normal tissues (*n* = 37). **h** Kaplan–Meier overall survival analyzed and compared between patients with high (LINC01111-high, *n* = 46) and low (LINC01111-low, *n* = 49) expression of LINC01111 in tumor.

Initially, we screened for lncRNAs that were aberrantly expressed in PC tissues comparing to normal pancreatic tissues. We selected 216 aberrantly expressed lncRNAs, of which 136 were downregulated and 80 were upregulated. Then, we screened for lncRNAs that were aberrantly expressed in plasma from PC patients vs. plasma from healthy people. We selected 72 aberrantly expressed lncRNAs, of which 34 were upregulated and 38 were downregulated; Fig. 1a provided an overview of the differentially expressed lncRNAs. We then analyzed the lncRNAs that were expressed at low levels in PC tissues and in the plasma of PC patients, and 4 lncRNAs were found to be simultaneously expressed at low levels. Among these selected lncRNAs, LINC01111 showed the lowest level in PC tissues and plasma of PC patients.

To explore the expression of LINC01111 in PC, qRT-PCR was performed, and we found that LINC01111 was significantly downregulated in PC cell lines and PC tissues (Fig. 1b, c). Additionally, we tested LINC01111 expression in human plasma; the results showed that LINC01111 level in plasma of PC patients was relatively lower than that in plasma of health control people (Fig. 1d). The results indicated that LINC01111 might play an important role in PC. As shown in Fig. 1e, LINC01111 was highly expressed in the cytoplasm according to the results of the fluorescence in situ hybridization (FISH) analysis of PC cells.

Further in situ hybridization (ISH) assays of LINC01111 were performed in 58 pairs of paraffin-embedded PC surgical specimens to measure the expression of LINC01111. Significant downregulation of LINC01111 was observed in PC tumor tissues compared with adjacent normal tissues (Fig. 1f, g). Notably, Kaplan–Meier survival analysis suggested that PC patients with higher tissue LINC01111 levels (*n* = 46; median survival of 29.2 months) had relatively increased overall survival (OS) compared with those with lower levels (*n* = 49; median survival of 13.9 months; Fig. 1h). Furthermore, we observed no significant relationship between LINC01111 expression and age, gender or tumor size, while the downregulation of LINC01111 expression was significantly associated with lymph node metastasis and tumor stage (Table 1).

**Table 1:** Relationship between LINC01111 expression detected by ISH and the clinical characteristics of 95 PC patients.

Clinical characteristics	*N*	LINC01111 expression		*χ*^2^	*P*-value
		Low	High		
All cases	95	49	46		
Age				0.848	0.357
<60	47	22	25		
≥60	48	27	21		
Gender				0.441	0.507
Male	59	32	27		
Female	36	17	19		
Tumor size (cm)				0.194	0.660
<5	62	33	29		
≥5	33	16	17		
Lymph node metastasis				4.867	0.027
Negative	53	22	31		
Positive	42	27	15		
TNM stage				5.957	0.015
I and II	65	28	37		
III and IV	30	21	9		

*P*-value < 0.05 was considered statistically significant. The *p* values were analyzed by a Pearson’s *χ*^2^ test using SPSS 22.0 software.

### LINC01111 inhibits cell proliferation in vitro and tumor growth in vivo

**Fig. 2:**
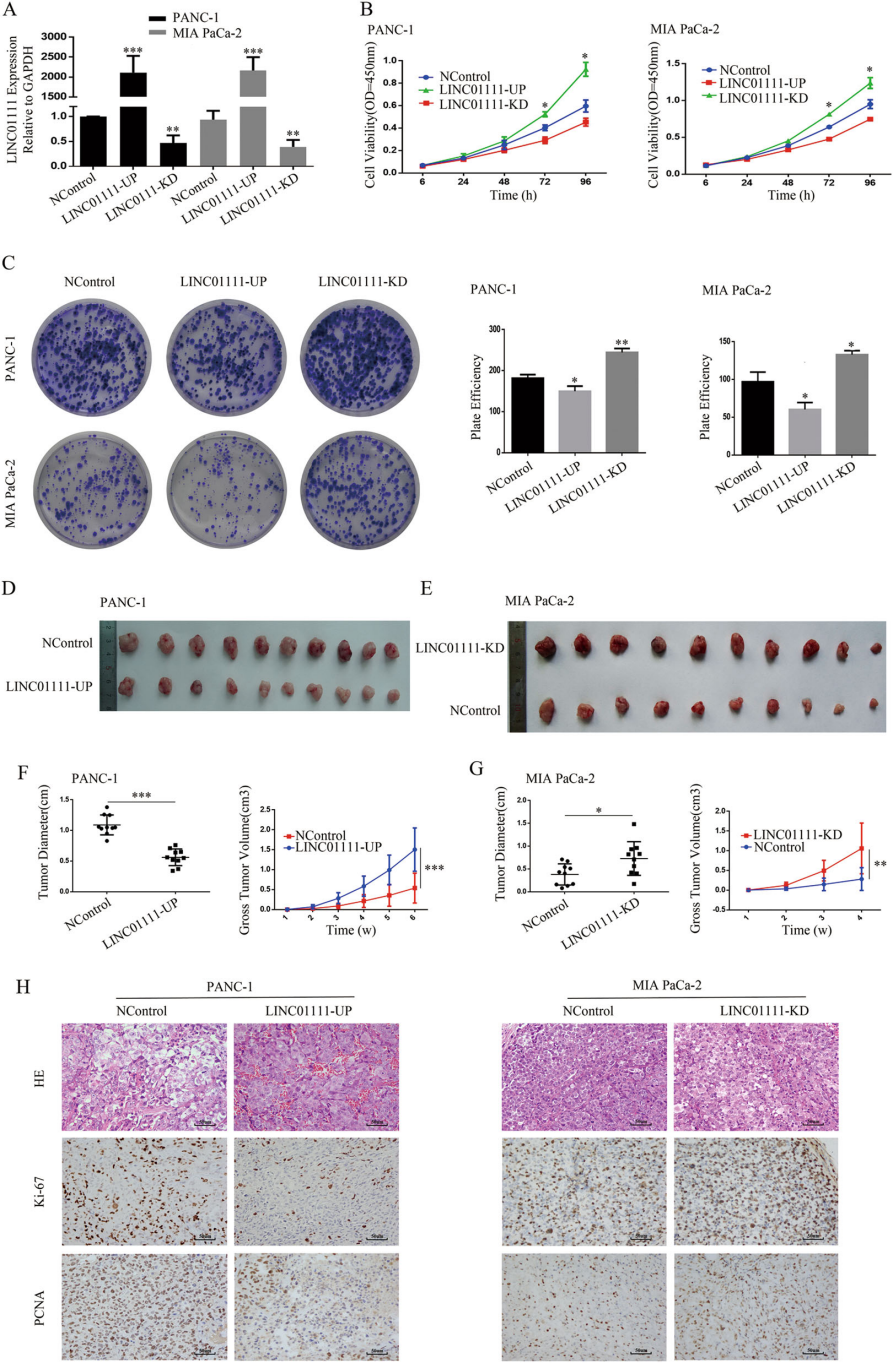
LINC01111 suppresses the PC cell growth in vitro and in vivo. **a** PCR analysis of LINC01111 expression in stable negative control (NControl), LINC01111-upregulated (LINC01111-UP) and LINC01111-knockdown (LINC01111-KD) PC cells. **p < 0.01, ***p < 0.001. **b** The CCK-8 assay comparing cell proliferation was performed in NControl, LINC01111-UP, and LINC01111-KD PC cells. All experiments were performed in triplicate, and data are presented as mean ± SD. *p < 0.05. **c** Representative images of colony formation assay (left panels) and analysis of colony numbers (right panels). All experiments were performed in triplicate, and data are presented as mean ± SD. *p < 0.05, **p < 0.01. **d** Images of tumors harvesting from the nude mice (10 per group) for PANC-1 cell line and **e** MIA PaCa-2 cell line. **f** Tumor volume at 6th week (left panels) and volume growth curve (right panels) of subcutaneous xenograft tumors for PANC-1 cell line. ***p < 0.001. **g** Tumor volume at 4th week (left panels) and volume growth curve (right panels) of subcutaneous xenograft tumors for MIA PaCa-2 cell line. Data are presented as mean ± SD. *p < 0.05, **p < 0.01. **h** Representative images of hematoxylin and eosin (HE) staining and IHC staining showing expression of protein Ki-67 and PCNA in various experimental groups of xenograft tumor tissues.

To investigate the biological function of LINC01111 in PC, we used a lentiviral system to establish stable LINC01111-upregulated (LINC01111-UP) and LINC01111-knockdown (LINC01111-KD) PC cells, and the qRT-PCR results confirmed the functioning system (Fig. 2a). The results of the CCK-8 assays and colony formation assays revealed that the overexpression of LINC01111 significantly inhibited the proliferative capacity of PC cells compared with that of negative control (NControl) cells, while the knockdown of LINC01111 enhanced the proliferation of PC cells relative to that of NControl cells (Fig. 2b, c). Furthermore, we examined the expression levels of PCNA and Ki-67, key regulatory genes involved in cell proliferation and overexpression of which indicated enhancement of cell proliferation^16^. The results of western blotting in PC cells showed that the overexpression of LINC01111 decreased the levels of PCNA and Ki-67 comparing to that of NControl cells, while the knockdown of LINC01111 increased the levels of PCNA and Ki-67 relative to that of NControl cells (Fig. 4a).

As shown in Fig. 1b, LINC01111 expression was relatively higher in the MIA PaCa-2 cell lines than in the PANC-1 cell lines. To explore the effect of LINC01111 on the tumorigenicity of PC cells in vivo, LINC01111-UP and NControl PANC-1 cells and LINC01111-KD and NControl MIA PaCa-2 cells were injected subcutaneously into the upper right flanks of nude mice. There were no obvious effects on the weight of the mice (Supplementary Fig. 1A, B). The tumor volume increased much more slowly, and the tumor weight was lower at the 6th week in the LINC01111-UP group compared with the NControl group (Fig. 2d, f), whereas the LINC01111-KD group showed the inverse results (Fig. 2e, g), indicating that LINC01111 overexpression remarkably inhibited the tumorigenic capability, while LINC01111 knockdown promoted the tumorigenic capability of PC cells in vivo.

We then performed immunohistochemistry (IHC) staining of the xenograft tumor samples. The results showed that expression of the Ki-67 and PCNA proteins were lower in xenograft tumors from the LINC01111-UP group than in those from the NControl group but were higher in xenograft tumors from the LINC01111-KD group than in those from the NControl group (Fig. 2h).

### LINC01111 inhibits cell cycle progression in PC cells

To further reveal the possible mechanism involved in the modulation of PC cell proliferation by LINC01111, we measured cell cycle distribution by EdU incorporation assays and fluorescence-activated cell sorting (FACS). The results showed that EdU-positive cells, which represented mitotic S phase cells, were significantly decreased in PC cells with the overexpression of LINC01111. In contrast, the suppression of LINC01111 increased the number of EdU-positive cells (Supplementary Fig. 2A). As expected, the results of the cell cycle analysis by FACS were in accordance with those of the EdU incorporation assays (Supplementary Fig. 2B). Additionally, to confirm the biological functions of LINC01111 described above, we examined the expression levels of cyclin D1 and cyclin A2, which act as key regulators involved in the cell cycle progression and can promote G1/S transition^17,18^. The results of western blotting showed that the overexpression of LINC01111 decreased the levels of cyclin D1 and cyclin A2 comparing to that of NControl cells, while the knockdown of LINC01111 increased the levels of cyclin D1 and cyclin A2 (Fig. 4a). Collectively, these data suggest the role of LINC01111 in blocking the G1/S transition.

### LINC01111 inhibits cell invasion and migration in vitro and tumor metastasis in vivo

**Fig. 3:**
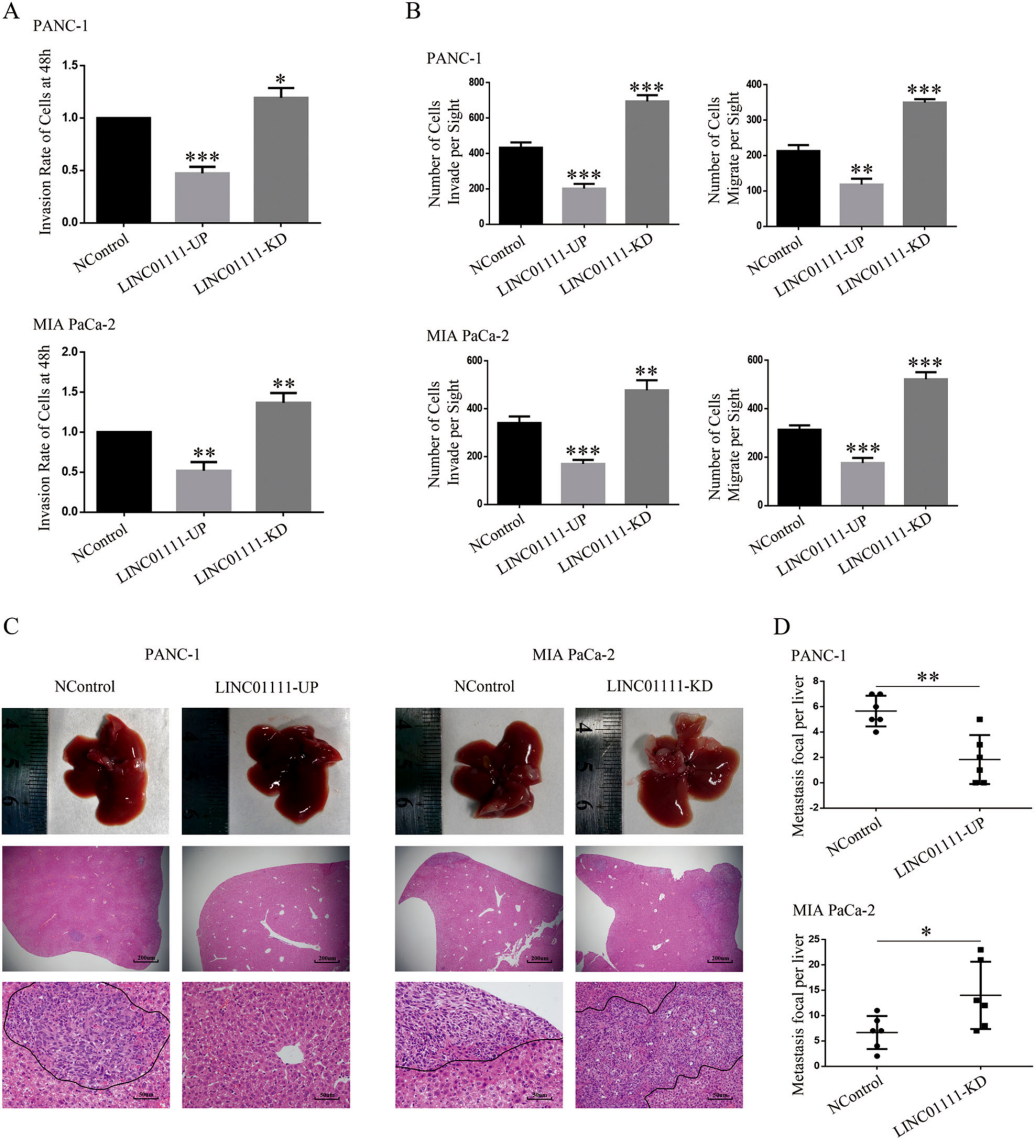
LINC01111 suppresses the PC cell invasion and migration in vitro and in vivo. **a** The bar graph showing the wound healing percentages of the indicated cell lines. All experiments were performed in triplicate, and data are presented as mean ± SD. *p < 0.05, **p < 0.01, ***p < 0.001. **b** The bar graph generated from the Transwell assay showing the invasion and migration of the indicated cell lines. All experiments were performed in triplicate, and data are presented as mean ± SD. **p < 0.01, ***p < 0.001. **c** Representative images of the liver metastasis models at 4th week. **d** The average numbers of visible metastatic nodules in the livers. Data are presented as mean ± SD. *p < 0.05, **p < 0.01.

**Fig. 4:**
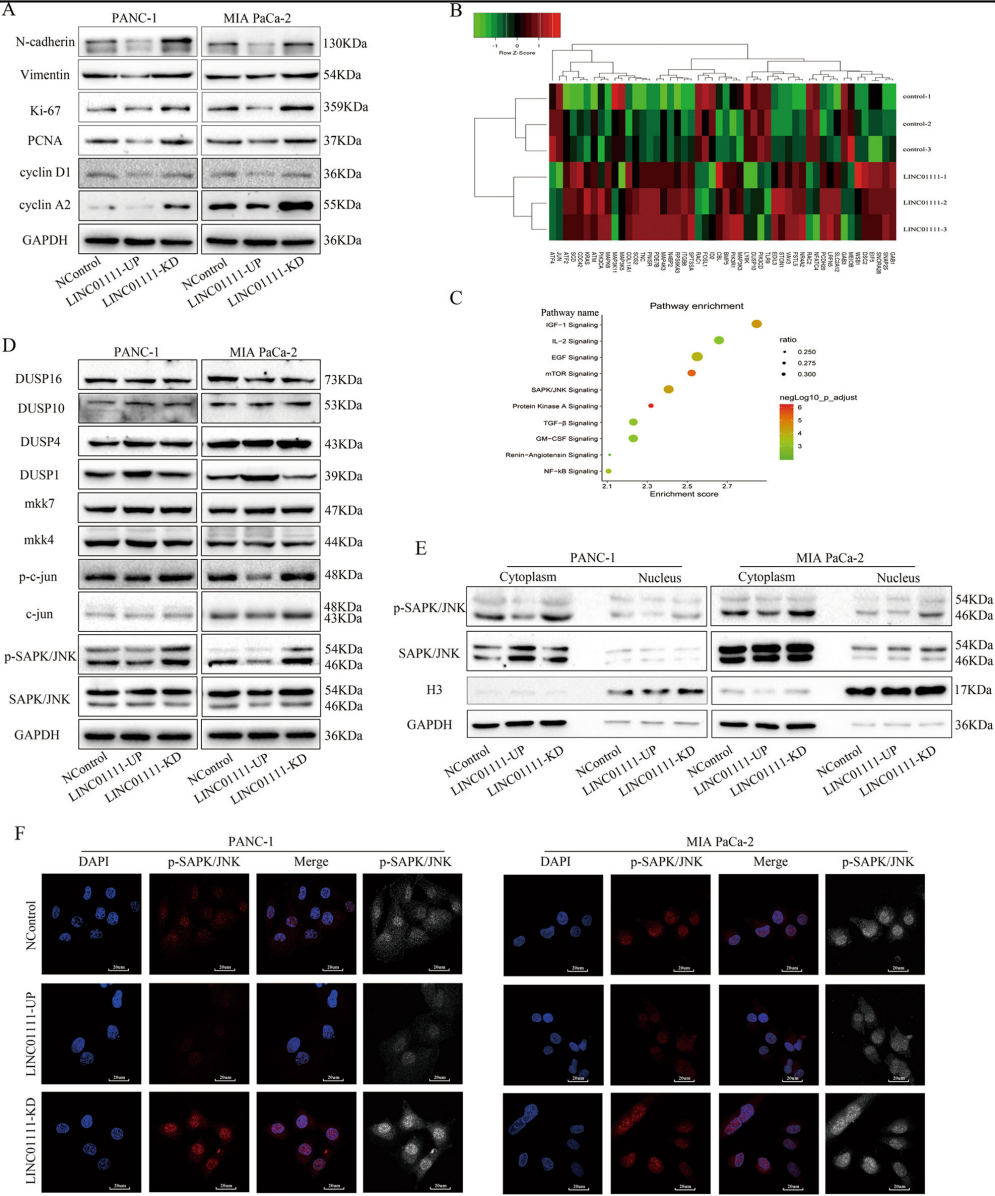
LINC01111 modulates the expression of several genes involved in cell proliferation, the cell cycle, cell invasion, and migration through SAPK/JNK signaling pathway in PC cells. **a** Western blotting showing protein expression involved in cell proliferation, the cell cycle, and metastasis in PC cells. **b** Hierarchical clustering analysis of microarray for PC cells (overexpressing LINC01111 and control cells) shows that LINC01111 could regulate genes involved in the SAPK/JNK signaling pathway. **c** Pathway enrichment analysis showing gene expression of subsets of genes related to the SAPK/JNK signaling pathway. **d** Western blotting showing the expression change of proteins involved in the SAPK/JNK signaling pathway. **e** Western blotting showing the abundance change of p-SAPK/JNK in the nucleus and the cytoplasm. **f** Representative confocal images showing the abundance change of p-SAPK/JNK in the nucleus and the cytoplasm.

We observed an inverse relationship between the level of LINC01111 and the TNM stage of PC, which prompted us to investigate whether LINC01111 could affect the invasion and migration of PC cells. Scratch wound healing assays revealed that the overexpression of LINC01111 reduced the invasive capability of PC cells compared with that of control cells, whereas the knockdown of LINC01111 promoted the invasion of PC cells (Fig. 3a and Supplementary Fig. 3A). Furthermore, Transwell assays with or without Matrigel showed that LINC01111 had the same effect on PC cell invasion and migration (Fig. 3b and Supplementary Fig. 3B). Researches revealed that the expression level of N-cadherin and Vimentin could indicate the capacity of invasion and metastasis of cancer cells^19^. We then examined the expression levels of N-cadherin and Vimentin by western blotting in PC cells, and the results showed that the overexpression of LINC01111 decreased their levels while the downregulation of LINC01111 increased their expression (Fig. 4a).

Next, we injected stable LINC01111- upregulated and LINC01111-knockdown PC cells into the spleens of nude mice to generate a spleen xenograft tumor model. After 4 weeks, we killed the model mice. To histologically examine the liver metastases, the livers of the mice were collected for HE staining. The results showed that the LINC01111-UP PANC-1 cells generated fewer and smaller live colonies than cells from the NControl group, whereas LINC01111-KD MIA PaCa-2 cells generated more and larger colonies than cells from the NControl group. Moreover, HE-stained liver sections revealed that the control tumors maintained distinct tumor–stroma boundaries, whereas the LINC01111-KD tumors showed extensive evidence of invasion into the adjacent tissue (Fig. 3c, d).

As the IHC staining results showed, E-cadherin expression in xenograft tumors was higher in the LINC01111-UP group than in the NControl group but lower in the LINC01111-KD group compared with the NControl group. As expected, the N-cadherin expression pattern was reversed (Supplementary Fig. 4). Overall, these data suggest that LINC01111 inhibits cell invasion and migration in vitro and tumor metastasis in vivo.

### LINC01111 can modulate the SAPK/JNK signaling pathway

To gain a better understanding of the molecular mechanism by which LINC01111 exerted its tumor suppressive function in PC cells, we performed mRNA microarray analysis to analyze the effects of LINC01111 overexpression on the gene expression profile. The results of gene ontology analysis and Gene Ontology (GO) analyses indicated the significant enrichment of genes involved in the SAPK/JNK signaling pathway (Fig. 4b, c). We confirmed the regulatory effects of LINC01111 on the expression changes of genes in the SAPK/JNK signaling pathway by western blotting of PC cells (Fig. 4d). The results also indicated that the overexpression of LINC01111 could decrease the phosphorylation level of SAPK/JNK, while the knockdown of LINC01111 had the opposite effect.

Next, we investigated the phosphorylation level of the SAPK/JNK protein by western blotting and immunofluorescence in LINC01111-UP, LINC01111-KD and NControl PC cells. As shown in the illustrations (Fig. 4e, f), the phosphorylation of SAPK/JNK was decreased both in the nucleus and cytoplasm in the LINC01111-UP group compared with the NControl group, while the knockdown of LINC01111 expression reversed these effects. These data indicated that the SAPK/JNK signaling pathway was the targeted signaling pathway through which LINC01111 exerted its tumor suppressive effects in PC cells.

### Overexpression of LINC01111 can rescue the effects of SAPK/JNK inhibition on PC cells

**Fig. 5:**
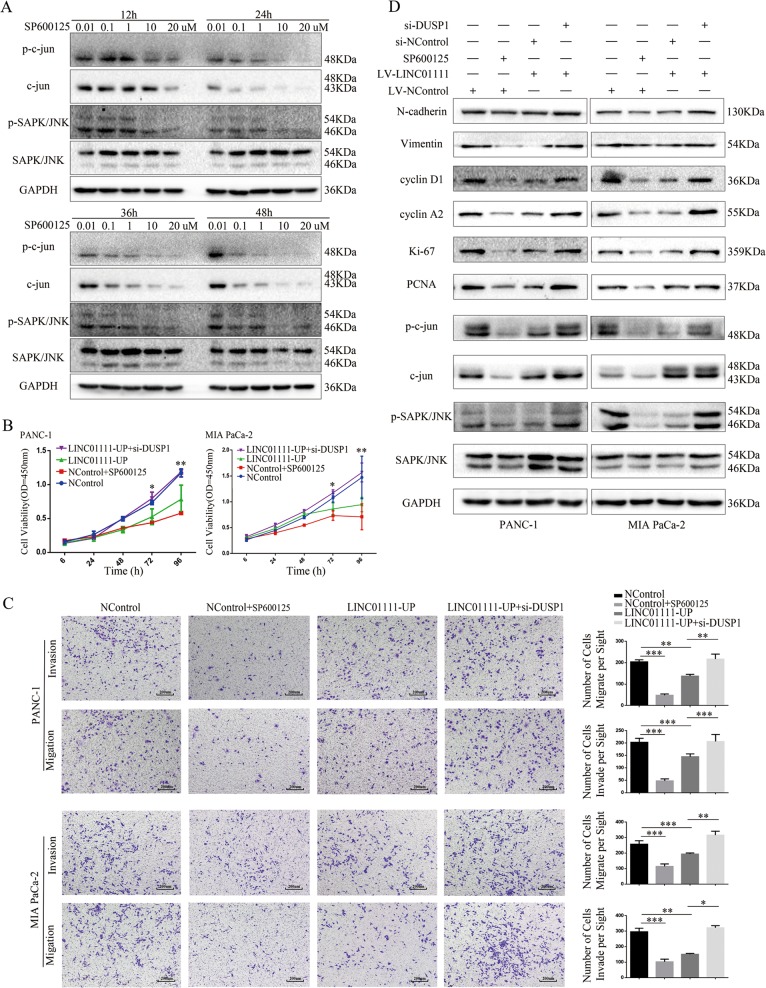
Overexpression of LINC01111 can rescue the effects of PC cells induced by SPAK/JNK inhibition. **a** The effect of the SAPK/JNK signaling pathway inhibitor SP600125 detected by western blotting. **b** The CCK-8 assay comparing cell proliferation was performed in PC cells when treated differently (NControl, NControl + SP600125, LINC01111-UP, LINC01111-UP+si-DUSP1). All experiments were performed in triplicate, and data are presented as mean ± SD. *p < 0.05, **p < 0.01. **c** Transwell assay evaluating cell invasion and migration was performed in PC cells when treated differently. All experiments were performed in triplicate, and data are presented as mean ± SD. *p < 0.05, **p < 0.01, ***p < 0.001. **d** Western blotting showing the expression change of proteins involved in the SAPK/JNK signaling pathway when treated differently.

Interestingly, we found that DUSP1 expression was greatly upregulated by the overexpression of LINC01111 and inhibited by the knockdown of LINC01111 (Fig. 4d). Studies have defined DUSP1 as a dual-specificity phosphatase that dephosphorylates and inactivates mitogen-activated protein kinase (MAPK) both in vitro and in vivo^20^. Thus, we proposed that DUSP1 is a key downstream target of LINC01111. To confirm our hypothesis, we inhibited the SAPK/JNK signaling pathway using the pathway inhibitor SP600125 and downregulated the DUSP1 expression level with siRNA in PC cells. As shown in Fig. 5a, SP600125 treatment of PANC-1 cells inhibited the SAPK/JNK signaling pathway in a dose- and time-dependent manner, and the results of western blotting suggested that treatment with 10 µM SP600125 for 36 h generated the optimal effect. The results of the CCK-8 assay showed that SP600125 could inhibit the proliferation of PC cells, and the overexpression of LINC01111 generated the same effect. Furthermore, the downregulation of DUSP1 by siRNA restored the inhibition of cell proliferation caused by SAPK/JNK inhibition and LINC01111 upregulation (Fig. 5b). Transwell assays with or without Matrigel showed that LINC01111 and DUSP1 had the same effects on PC cell invasion and migration (Fig. 5c). Moreover, western blotting showed the same results (Fig. 5d). Overall, LINC01111 could modulate the SAPK/JNK signaling pathway via DUSP1 in PC cells.

### LINC01111 upregulates its parental gene DUSP1 by sequestering miR-3924

**Fig. 6:**
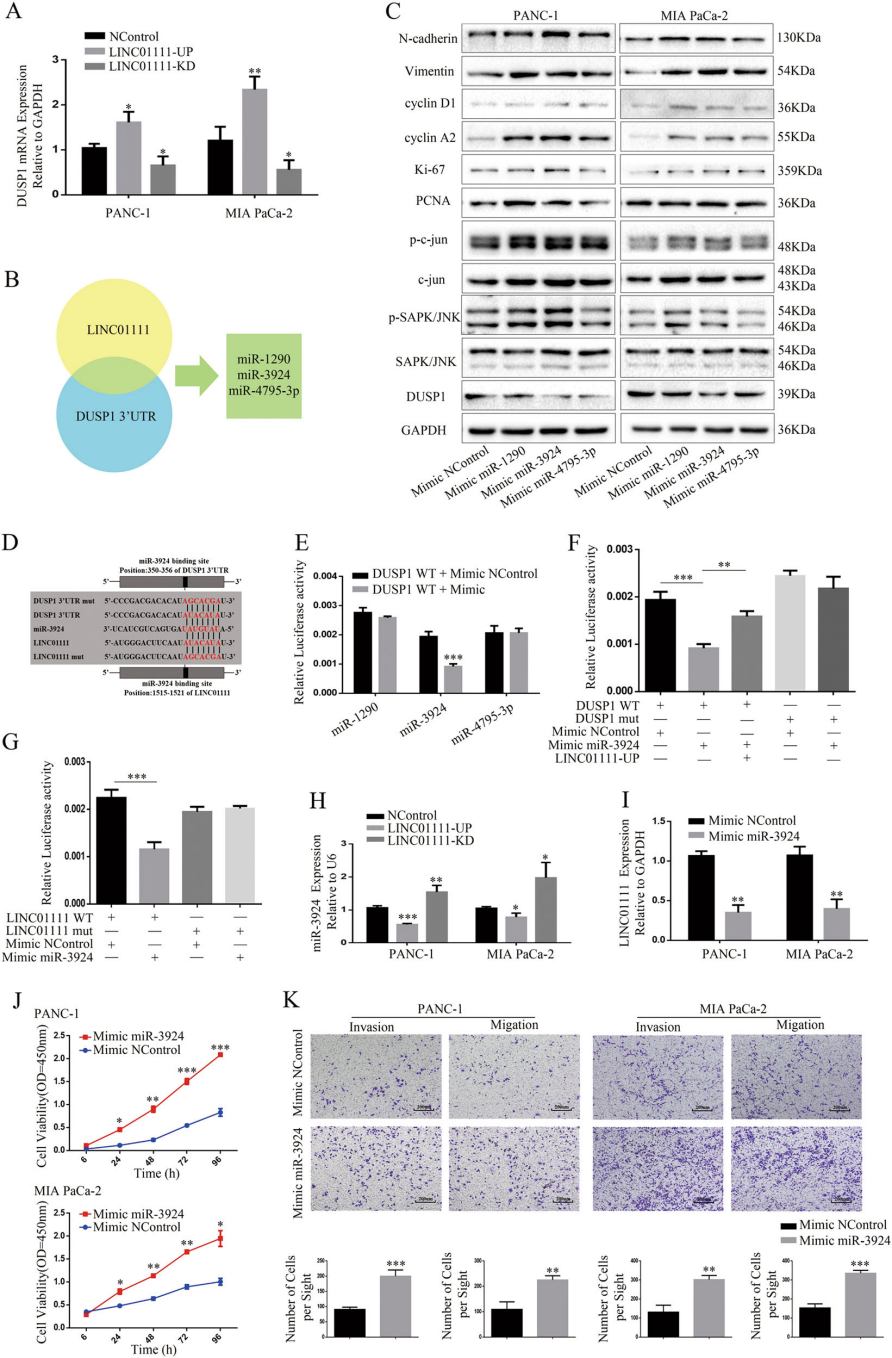
LINC01111 regulates proliferation, cell cycle and metastasis of PC cells via the modulation of DUSP1 expression by sequestering miR-3924. **a** PCR analysis showed expression change of DUSP1 mRNA with augment or attenuation of LINC01111 expression in PC cells. *p < 0.05, **p < 0.01. **b** The Venn diagram identified three microRNAs (miR-1290, miR-3924, and miR-4795-3p) targeting LINC01111 and DUSP1 3′UTR. **c** Western blotting showing the expression change of DUSP1 and proteins involved in the SAPK/JNK signaling pathway in PC cells with or without overexpression of target microRNAs (miR-1290, miR-3924, and miR-4795-3p). **d** Bioinformatics prediction showed binding sites of miR-3924 to DUSP1 3′UTR sequence using TargetScan and to LINC01111 sequence using sequence alignment. **e** Dual-luciferase assays showed that decreased luciferase activity was observed in HEK 293T cells when co-transfected with psi-CHECK-DUSP1-WT and Mimic-miR-3924, but not Mimic-miR-1290 or Mimic-miR-4795-3p. ***p < 0.001. **f** Dual-luciferase assays implied that decreased luciferase activity was observed in HEK 293T when co-transfected with psi-CHECK-DUSP1-WT and Mimic-miR-3924, and rescued luciferase activity occurred when LINC01111 was overexpressed, whereas co-transfection of psi-CHECK-DUSP1-mut and Mimic-miR-3924 had no influence on luciferase activity. **p < 0.01, ***p < 0.001. **g** Dual-luciferase assays implied that decreased luciferase activity was observed in HEK 293T cells when co-transfected with psi-CHECK-LINC01111-WT and Mimic-miR-3924 comparing to co-transfection of psi-CHECK-LINC01111-mut and Mimic-miR-3924. ***p < 0.001. **h** PCR analysis showed expression change of miR-3924 with augment or attenuation of LINC01111 expression in PC cells. *p < 0.05, **p < 0.01, ***p < 0.001. **i** Overexpression of miR-3924 decreased the LINC01111 level in PC cells quantified by qRT-PCR. **p < 0.01. **j** The CCK-8 assay showed the cell proliferation of PC cells with or without overexpression of miR-3924. *p < 0.05, **p < 0.01, ***p < 0.001. **k** Transwell assay evaluating cell invasion and migration ability was performed in PC cells with or without overexpression of miR-3924. **p < 0.01, ***p < 0.001. All experiments were performed in triplicate, and data are presented as mean ± SD.

The above experiments demonstrated that the aberrant expression of LINC01111 might be involved in gene control and oncogenic function. Thus, we further investigated whether LINC01111 could affect DUSP1 expression as parts of its important role in growth and progression of PC. As LINC01111 localized predominantly in the cytoplasm of PC cells according to the results of the FISH analysis (Fig. 1e), it might function as a ceRNA and sequester corresponding microRNAs, which leads to the liberation of microRNA-targeted gene transcripts. Moreover, we found that the downregulation of LINC01111 could reduce the abundance of DUSP1 in PC cells at both the mRNA level (Fig. 6a) and protein level (Fig. 4d). Based on the cytoplasm location of LINC01111 and the synchronous expression change of LINC01111 and DUSP1, we hypothesized that LINC01111 played a role as a ceRNA to regulate the expression of DUSP1.

To demonstrate our hypothesis, we used a bioinformatics analysis to reveal potential sites through which miRNAs targeting the DUSP1 3′ UTR (TargetScan, http://www.targetscan.org/cgi-bin/targetscan/vert_72/view_gene.cgi?rs=ENST00000239223.3&taxid=9606&members=miR-3924&showcnc=1&shownc=1&shownc_nc=&showncf1=1&showncf2=&subset=1) and miRNAs targeting LINC01111 (through sequence alignment, http://www.mirdb.org/cgi-bin/custom_predict/customDetail.cgi). Based on these results, we identified three potential miRNAs, miR-1290, miR-3924, and miR-4795-3p (Fig. 6b). Next, the results of western blotting suggested that all three miRNAs promoted the proliferation and metastasis of PC cells; among them, miR-3924 showed the most obvious effect (Fig. 6c). Furthermore, we performed a luciferase activity assay to test whether the three miRNAs were able to influence the expression of DUSP1. As shown in Fig. 6d and Fig. 6e, the luciferase reporter assay revealed that the wild-type 3′ UTR of DUSP1 resulted in decreased luciferase activity when mimics of miR-3924 but not miR-1290 and miR-4795-3p were added, which suggested that miR-3924 but not miR-1290 and miR-4795-3p acted as a negative regulator of DUSP1. Moreover, the overexpression of LINC01111 could rescue the decrease in luciferase activity caused by the binding of mimics of miR-3924 to the wild-type 3′ UTR of DUSP1, while the mutated 3′ UTR of DUSP1 did not show a significant response to mimics of miR-3924 (Fig. 6f). In addition, the luciferase reporter assay showed that the overexpression of miR-3924 by using mimics could depress the luciferase activity of wild-type LINC01111, whereas the augmentation of miR-3924 had no effect on mutant LINC01111, indicating that LINC01111 can directly bind to miR-3924 (Fig. 6d, g). Furthermore, LINC01111 overexpression attenuated miR-3924 levels, while the knockdown of LINC01111 augmented miR-3924 levels (Fig. 6h). As expected, the expression of LINC01111 was apparently decreased after transfection with miR-3924 mimics (Fig. 6i). The CCK-8 assays and Transwell assays with or without Matrigel were used to explore the effects of miR-3924 on PC cells. The results showed that the overexpression of miR-3924 could promote cell proliferation, invasion, and migration of PC cells (Fig. 6j, k). Overall, these data indicate that LINC01111 regulates DUSP1 expression by sequestering miR-3924.

## Discussion

**Fig. 7:**
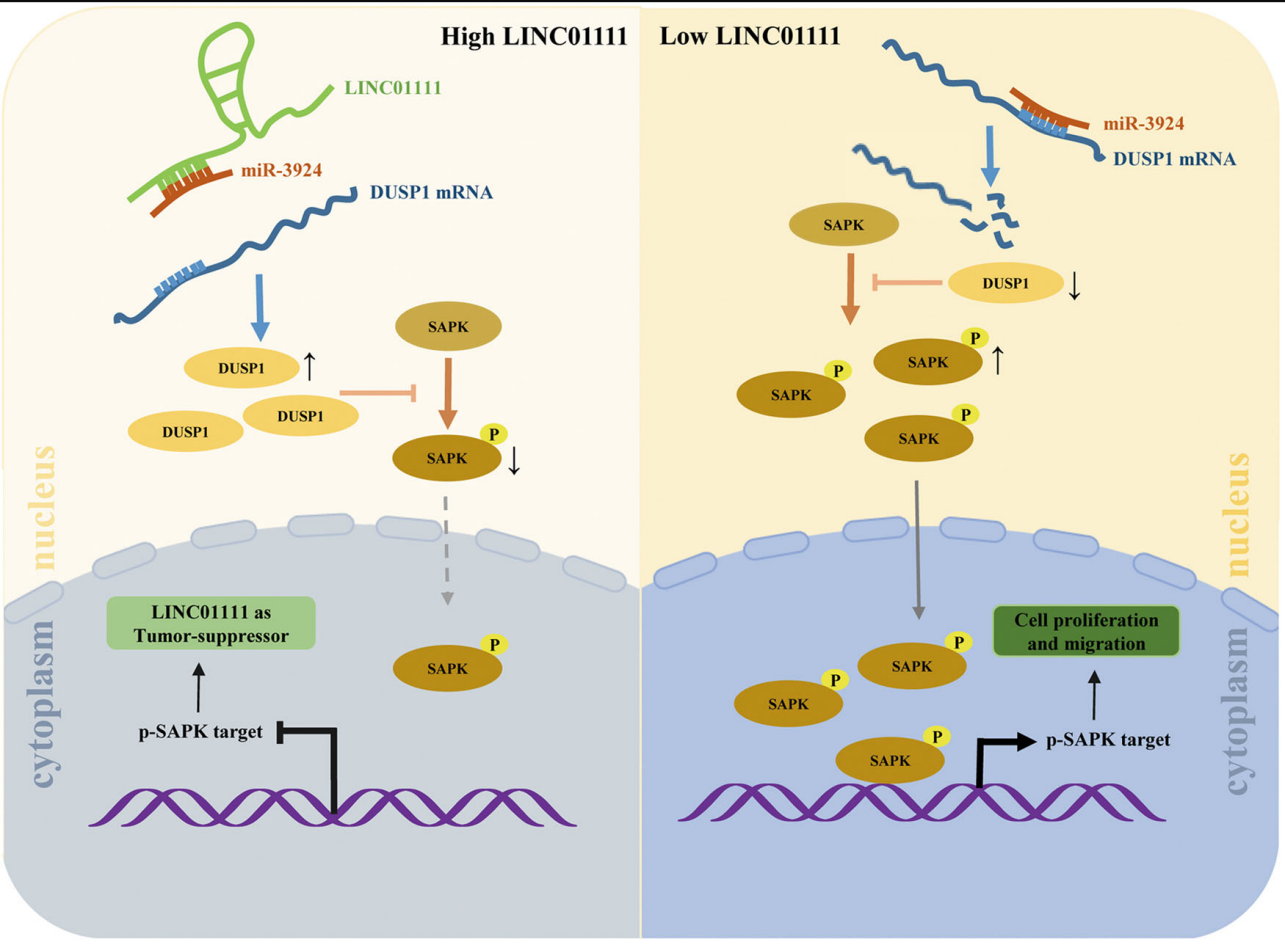
Schematic model shows the results of the study. LINC01111 functions through a ceRNA mechanism by competing with endogenous miR-3924, thus triggering DUSP1 protein expression in PC.

In the present study, we demonstrated that LINC01111 expression levels are clearly decreased and that the SAPK/JNK signaling pathway is significantly activated by the downregulation of DUSP1 protein in PC, which promotes tumorigenesis and tumor metastasis. Mechanistically, we found that LINC01111 functions through a ceRNA-involved mechanism by competing with endogenous miR-3924, thus triggering DUSP1 protein expression (Fig. 7). These findings revealed that LINC01111 acts as a key molecule involved in PC initiation and progression.

It is well accepted that lncRNAs play important roles in regulating biological and pathological processes, and many of them have been researched and reported. Some lncRNAs act as tumor promoters, which have been used as biomarkers or therapeutic targets for cancer treatment, such as HOTAIR^7^, MALAT1^21^, and H19^22^. Other lncRNAs, including TSLNC8^9^, MEG3^23^ and loc285194^24^, have recently been reported to function as tumor suppressors, which modulate tumorigenesis and cancer progression when their functions are lost.

In this study, results from the GEO database indicated that LINC01111 expression level was significantly decreased in PC tissues comparing to that in normal pancreatic tissues, and that the LINC01111 expression level was significantly lower in plasma of PC patients than that of healthy subjects. As expected, the detection of LINC01111 by qRT-PCR in PC tissues and plasma of patients found similar results. Furthermore, ISH analyses revealed significant downregulation of LINC01111 expression in 95 paired paraffin-embedded PC surgical specimens and that a low level of LINC01111 was associated with poor prognosis of PC patients, indicating that LINC01111 acts as a tumor suppressor in PC.

Cancer is a disease that involves dynamic changes in the genome, including alterations in DNA, RNA and proteins^25^. In biological processes, lncRNAs can interact with DNA, RNA, proteins, and/or combinations of these, resulting in chromatin organization regulation, transcriptional and post-transcriptional regulation, and cell signaling pathway regulation^26^. Changes in lncRNA expression trigger cancer cells to gain capacities for tumor initiation, growth, and metastasis^27–29^. We performed gain-of-function and loss-of-function experiments to further study the biological function of LINC01111 in PC cells. The results showed that the stable decreased expression of LINC01111 enabled PC cells to have higher capability for cell proliferation, enhancement of cell-cycle G1/S transition, higher tendency to invasion and migration in vitro, and promoted tumor growth and metastasis in vivo. Inversely, stable elevated LINC01111 expression generated the opposite effects. These data revealed the essential role of LINC01111 in PC.

The SAPK/JNK signaling pathway is related to the mitogen-activated protein kinase (MAPK) signaling cascade, involving the sequential phosphorylation and activation of the proteins MAPK/extracellular signal-regulated kinase (ERK) kinase kinase 1, SAPK/ERK kinase 1, SAPK/JNK, and c-Jun^30,31^. Phosphorylation activates function of SAPK/JNKs, which subsequently translocate from the cytoplasm to the nucleus, where they function and phosphorylate series of genes including c-Jun, ATF2, etc. and enhance their transcriptional activity^16^. As reported, c-Jun can positively regulate expression of proliferation-related genes including cyclin D1 and cyclin A2^16,17,32^, and genes involved in cell invasion and migration, including N-cadherin and vimentin^33,34^. Previous studies revealed the important role of the SAPK/JNK signaling pathway in regulating cell survival, proliferation, and apoptosis^35,36^, and its critical role in the chemotherapeutic treatment of human cancer^37,38^. In the present study, we performed mRNA microarray analysis to analyze the gene expression profile affected by LINC01111 overexpression. According to the results, we found a significant enrichment of genes involved in the SAPK/JNK signaling pathway, which made the SAPK/JNK signaling pathway our target of interest. Western blotting showed that the knockdown of LINC01111 in PC cells activated the SAPK/JNK signaling pathway, whereas LINC01111 overexpression inhibited it. Furthermore, according to the immunofluorescence analyses of PC cells, the knockdown of LINC01111 increased SAPK phosphorylation in the cytoplasm and the nucleus, whereas LINC01111 overexpression generated the inverse. It remains unknown how LINC01111 functions as a SAPK/JNK inhibitor.

Interestingly, we observed the coexpression of DUSP1 protein and LINC01111 when conducting western blotting. DUSP1, first identified ~30 years ago, is a member of the threonine-tyrosine dual-specificity phosphatase family, the function of which is to dephosphorylate and therefore inactivate the MAP kinases, including ERKs, p38 MAPKs, and JNKs^14,39^. Previous studies revealed that DUSP1 may play opposing roles in different cancer types or different stages of cancer. In the early phase of cancer, the upregulation of DUSP1 enables tumors to evade SAPK-induced death, while in the advanced stages of cancer, the downregulation of DUSP1 promotes cell proliferation, increases tumor growth, and actuates the metastatic process^39–44^. In this study, we demonstrated that DUSP1 was downregulated by LINC01111 knockdown, which inactivated the SAPK/JNK signaling pathway in the progression of PC.

Currently, the regulatory mechanisms of ncRNAs are becoming more deeply understood. As reported, some lncRNAs act as molecular sponges to sequester target miRNAs and regulate their function^45,46^. In the present study, bioinformatics analysis indicated potential binding sites in LINC01111 and miR-3924, as well as miR-3924 and DUSP1 3′ UTR, suggesting the possibility that LINC01111acts as a molecular sponge for miR-3924 to regulate the expression level of DUSP1. Next, we conducted experiments to demonstrate our hypothesis. The data showed that, similar to miR-3924 mimics, LINC01111 downregulation could inhibit expression of DUSP1, target gene of miR-3924, whereas LINC01111 upregulation inhibited the function of miR-3924, leading to increased levels of DUSP1. Therefore, the effect of LINC01111 on PC initiation and progression can be partly explained with a ceRNA mechanism, through which LINC01111 functions as a tumor suppressor.

In conclusion, we found that the lncRNA LINC01111 was downregulated in PC tissues and plasma of PC patients, and was characterized as a novel tumor suppressor. The loss of LINC01111 was associated with tumor progression and metastasis, and the level of LINC01111 was positively correlated with prognosis of PC patients. Gain-of-function and loss-of-function experiments revealed that the overexpression of LINC01111 can reduce the malignancy of PC cells in vitro and in vivo. LINC01111 can function as a molecular sponge for miR-3924 to upregulate DUSP1 protein levels and then downregulate SAPK phosphorylation and the translocation of p-SAPK from the cytoplasm to the nucleus. Thus, the loss of LINC01111 in PC activates the SAPK/JNK signaling pathway, resulting in the promotion of tumor progression and metastasis. LINC01111 may serve as a prognostic predictor for PC patients, and the LINC01111/miR-3924/DUSP1 axis is a potential therapeutic target for treating PC.

## Supplementary information

Reproducibility checklist

Attribution of authorship

Supplementary Table 1

Supplementary Table 2

Supplementary figure legends

Supplementary figure 1

Supplementary figure 2

Supplementary figure 3

Supplementary figure 4
