# Supplementary material for: Long noncoding RNA LINC01111 suppresses pancreatic cancer aggressiveness by regulating DUSP1 expression via microRNA-3924
Source: Cell Death Dis. 2019 Nov 25;10(12):883. doi: 10.1038/s41419-019-2123-y (PMC6877515; doi:10.1038/s41419-019-2123-y)
Supplement: Supplementary file 2 — Attribution of authorship [file 41419_2019_2123_MOESM2_ESM.pdf]

DECLARATION OF CONTRIBUTIONS TO ARTICLE

ADMC

Manuscript Number: CDDIS-19-2574R

Journal Name: *Cell Death & Disease* (the 'Journal')

Proposed Title of the Contribution: Attribution of authorship (the 'Contribution)

Author(s): Shutao Pan, Ming Shen, Min Zhou, Xiuhui Shi, Ruizhi He, Taoyuan Yin, Min Wang, Xingjun Guo, Renyi Qin (the 'Authors')

For all *CDDis* articles, each person named as an author in the published version must be able to show he or she has contributed substantially to the article.

Authorship credit should be based on 1) substantial contributions to conception and design, acquisition of data, or analysis and interpretation of data; 2) drafting the article or revising it critically for important intellectual content; and 3) final approval of the version to be published. Authors should meet conditions 1, 2 and 3.

Any person who cannot be shown to have made a substantial contribution to the article cannot be listed as an author in the final version. The name of any person who is deemed to have made a minor contribution can, however, appear in the Acknowledgments section of the article.

Please complete the table below to indicate the contributions of all named authors to the manuscript.

Author Full Name:	Specification of Contribution to the Manuscript:
Shutao Pan	Conceptualization, Data curation, Investigation, Writing–original draft
Ming Shen	Conceptualization, Investigation, Methodology
Min Zhou	Software, Investigation
Xiuhui Shi	Supervision, Visualization
Ruizhi He	Software
Taoyuan Yin	Formal analysis
Min Wang	Supervision, Visualization
Xingjun Guo	Conceptualization, Funding acquisition, Methodology, Project administration, Writing–review & editing
Renyi Qin	Funding acquisition, Methodology, Project administration, Writing–review & editing

Please complete the table below to indicate the contributions of all named authors to the figures.

Figure 1:

GXJ generated the data and prepared panel A; PST generated the qRT-PCR data and prepared panel B, C, and D; PST and SM generated the FISH and ISH data and labelled the image of panel E, F, G, and H; SXH and YTY analysed the data; ZM and HRZ assembled the figure.

Figure 2:

PST conducted the experiments and prepared panel A, B, and C; PST and ZM carried out the animal experiments and generated the data and labelled the image of panel D, E, F, G, and H; SXH and YTY analysed the data; ZM and HRZ assembled the figure.

Figure 3:

PST and SM conducted the experiments and prepared panel A and B; PST and ZM carried out the animal experiments and generated the data and labelled the image of panel C and D; SXH and YTY analysed the data; ZM and HRZ assembled the figure.

Figure 4:

PST and SM conducted the experiments and prepared panel A, B, C and D; PST generated the immunofluorescence data and labelled the image of panel E; SXH and YTY analysed the data; ZM and HRZ assembled the figure.

Figure 5:

PST and SM conducted the experiments and prepared panel A, B, C and D; SXH and YTY analysed the data; ZM and HRZ assembled the figure.

Figure 6:

PST generated the qRT-PCR data and prepared panel A, H and I; WM generated the data and prepared panel B and D; PST and SM generated the data and prepared panel C, E, F and G; PST generated the data and prepared panel J and K; SXH and YTY analysed the data; ZM and HRZ assembled the figure.

Signed for and on behalf of the Author(s):	Print Name:	Date:
	Renyi Qin	15 Jul. 2019
