# Supplementary material for: Long noncoding RNA LINC01111 suppresses pancreatic cancer aggressiveness by regulating DUSP1 expression via microRNA-3924
Source: Cell Death Dis. 2019 Nov 25;10(12):883. doi: 10.1038/s41419-019-2123-y (PMC6877515; doi:10.1038/s41419-019-2123-y)
Supplement: Supplementary file 3 — Supplementary Table 1 [file 41419_2019_2123_MOESM3_ESM.docx]

Supplemental Table 1. Primer sequences used for PCR

| Gene | Forward primer(5'-3') | Reverse primer(5'-3') |
| --- | --- | --- |
| GAPDH | GGAGCGAGATCCCTCCAAAAT | GGCTGTTGTCATACTTCTCATGG |
| LINC01111 | CCTCCATCATGAACCTGCAA | TGAAGGTACTGAGGCCATGTG |
| DUSP1 | GGATACGAAGCGTTTTCGGC | AGAGGTCGTAATGGGGCTCT |
| DUSP4 | TGCAGAATTCGCCCTTACGA | CATCCACAATTCAGCGAGCG |
| DUSP10 | AAGCCGAGGAGAAAACTCTGG | GAGAGCTAAGAAGGGACGCC |
| DUSP16 | TCGCGTCTACTAGGGGTGAT | TGCCTCCTTTGGCAACTTGA |
| JNK1 | AGTTGGGTGCATCATGGGAG | AGAAAGTTGGCAATCTACAGCA |
| JNK2 | AGTGGGTTGCATCATGGGAG | ACTGAGAAGGAGTGGCGTTG |
| c-jun | CGGAGCATTACCTCATCCCG | GACTATACTGCCGACCTGGC |
| ATF | TGTGAATTCTGCCAGGCAAT | GGAGAGGAAGGACCTGGGAT |
| NFATc1 | CCAGTACCAGCGTTTCACCT | CAAGAAAGTTGGCAAGAAAAAGC |
| cdc42 | TAAGTGTGTTGTTGTGGGCG | CAGTAGTGGGACAGGAAGCAG |
| MKK4 | ACTTCGGCATCAGTGGACAG | ACTATTCTCGCCATGCGTGT |
| MKK7 | GTATGGAGCCTGGGCATCTC | TCCGTTCACAGTGTCTGTCG |
