# Supplementary material for: Long noncoding RNA LINC01111 suppresses pancreatic cancer aggressiveness by regulating DUSP1 expression via microRNA-3924
Source: Cell Death Dis. 2019 Nov 25;10(12):883. doi: 10.1038/s41419-019-2123-y (PMC6877515; doi:10.1038/s41419-019-2123-y)
Supplement: Supplementary file 4 — Supplementary Table 2 [file 41419_2019_2123_MOESM4_ESM.docx]

Supplemental Table 2. Primers and sequence used for Si-RNA transfection

| Gene | Forward | Reverse |
| --- | --- | --- |
| LINC01111#1 | CCATTTCTCTTCTCAGCTTdTdT | dTdTAAGCTGAGAAGAGAAATGG |
| #2 | GCTGCAGATGAAGAGCAATdTdT | dTdTATTGCTCTTCATCTGCAGC |
| #3 | CCTCTGAAACTCCTGTTATdTdT | dTdTATAACAGGAGTTTCAGAGG |
| DUSP1 #1 | GCTCCACTCAAGTCTTCTTdTdT | dTdTAAGAAGACTTGAGTGGAGC |
| #2 | CCAATTGTCCTAACCACTTdTdT | dTdTAAGTGGTTAGGACAATTGG |
| #3 | GCTGGTCCTTATTTATTTAdTdT | dTdTTAAATAAATAAGGACCAGC |
