# Supplementary material for: Long noncoding RNA LINC01111 suppresses pancreatic cancer aggressiveness by regulating DUSP1 expression via microRNA-3924
Source: Cell Death Dis. 2019 Nov 25;10(12):883. doi: 10.1038/s41419-019-2123-y (PMC6877515; doi:10.1038/s41419-019-2123-y)
Supplement: Supplementary file 5 — Supplementary figure legends [file 41419_2019_2123_MOESM5_ESM.docx]

Supplementary Figure 1. LINC01111 suppresses the PC cell growth in vivo. (A) Weight of the mice at 6th week for the xenograft model for PANC-1 cell line. (B) Weight of the mice at 4th week for the xenograft model for MIA PaCa-2 cell line.

Supplementary Figure 2. LINC01111 suppresses the cell-cycle progression in PC cells. (A) EdU immunofluorescence staining showing the cell proliferation of PANC-1 and MIA PaCa-2 cells. The bar graph on the right shows the percentage of EdU-positive nuclei. All experiments were performed in triplicate, and data are presented as mean ± SD. *p < 0.05, ***p < 0.001. (B) FACS analysis of PANC-1 and MIA PaCa-2 cells with LINC01111 overexpression or knockdown. The bar graph on the right shows proportion of cells in various phases. All experiments were performed in triplicate, and data are presented as mean ± SD. *p < 0.05, **p < 0.01.

Supplementary Figure 3. LINC01111 suppresses the PC cell invasion and migration in vitro. (A) Invasion of the indicated cell lines evaluated by scratch wound healing assay. Images were analyzed using Image-pro plus 6.0. (B) Invasion and migration of the indicated cell lines evaluated by Transwell assay with and without matrigel.

Supplementary Figure 4. LINC01111 suppresses the PC cell invasion and migration in vivo. (A) Representative images of HE and IHC staining showing protein E-cadherin and N-cadherin expression in xenograft tumor tissues from the various experimental mouse groups.
