# Supplementary figures and images for: Long noncoding RNA LINC01111 suppresses pancreatic cancer aggressiveness by regulating DUSP1 expression via microRNA-3924
Source: Cell Death Dis. 2019 Nov 25;10(12):883. doi: 10.1038/s41419-019-2123-y (PMC6877515; doi:10.1038/s41419-019-2123-y)

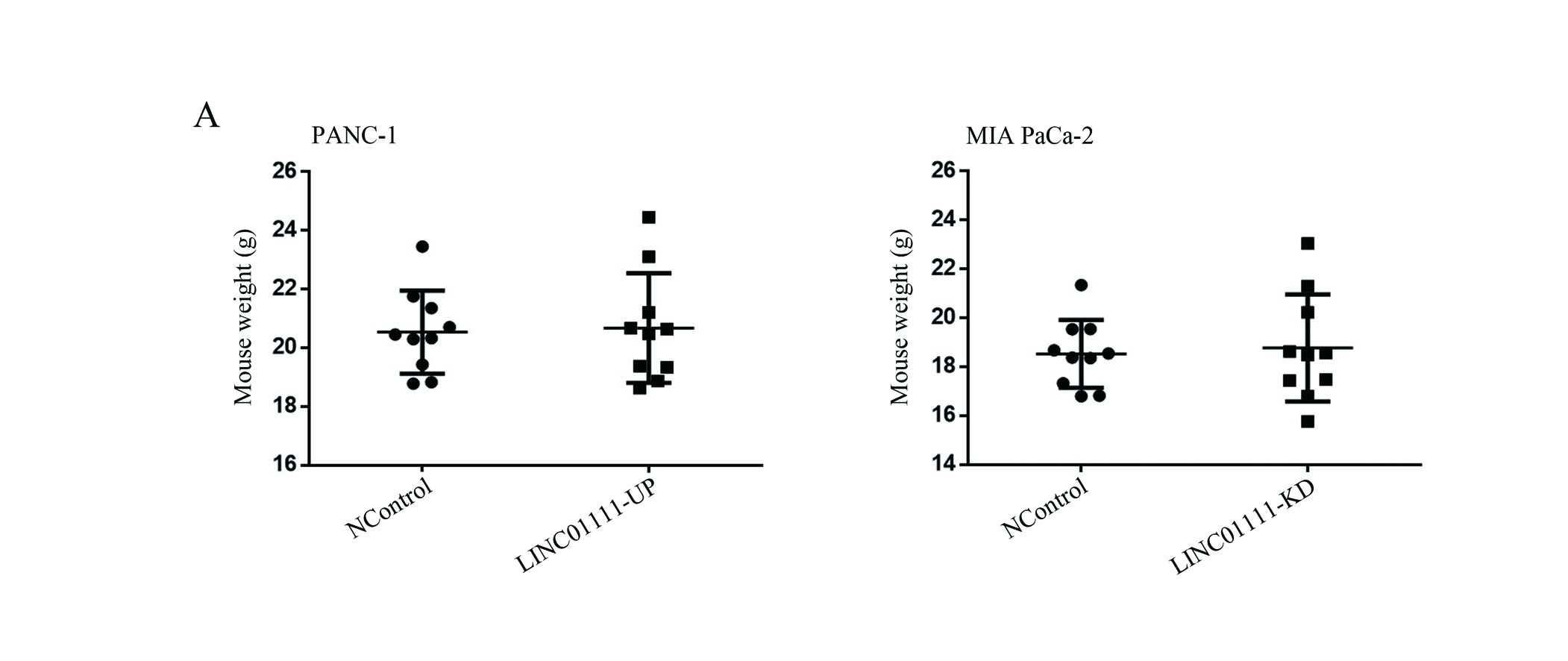

Supplement: Supplementary file 6 — Supplementary figure 1 [file 41419_2019_2123_MOESM6_ESM.tif]

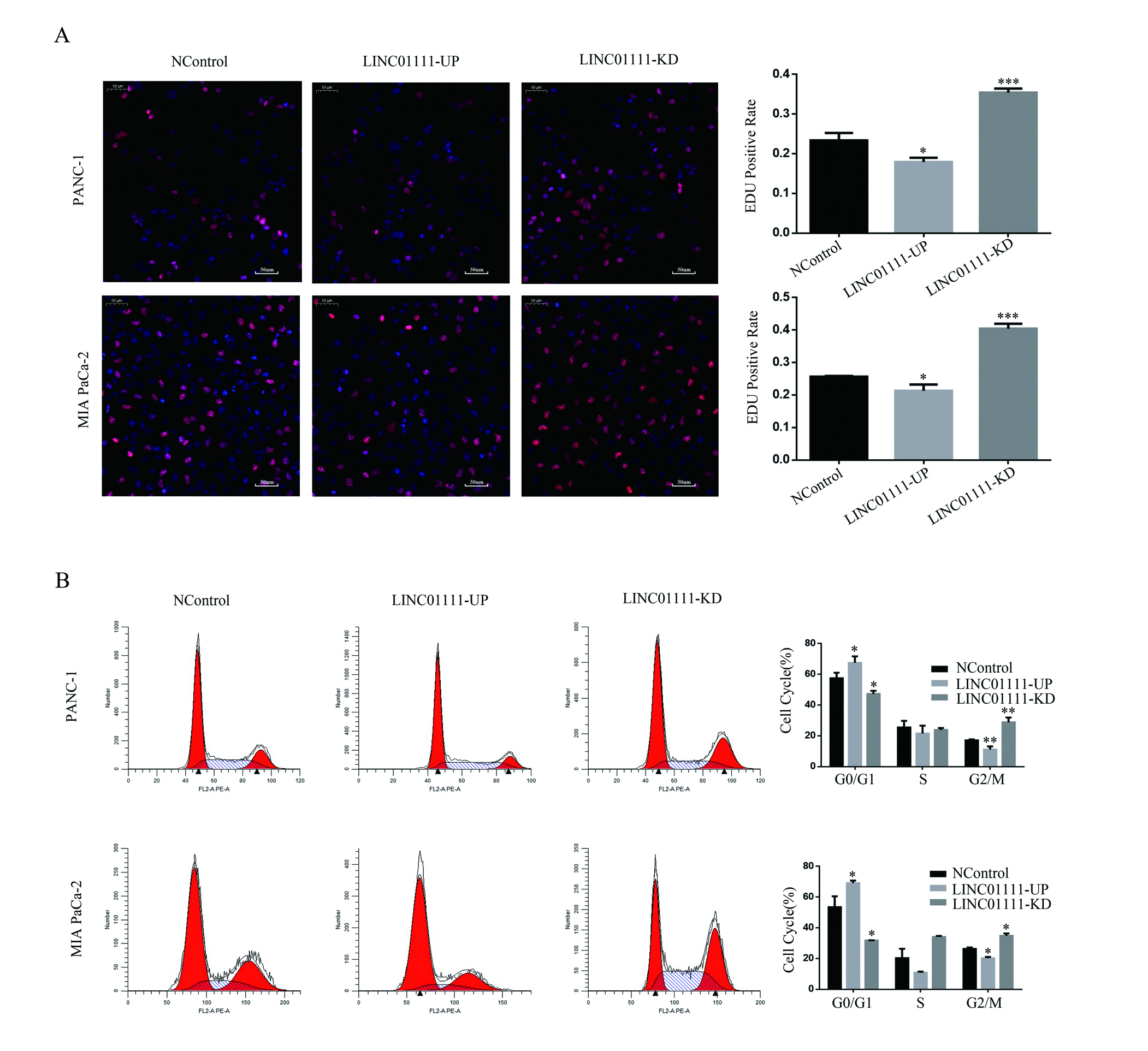

Supplement: Supplementary file 7 — Supplementary figure 2 [file 41419_2019_2123_MOESM7_ESM.tif]

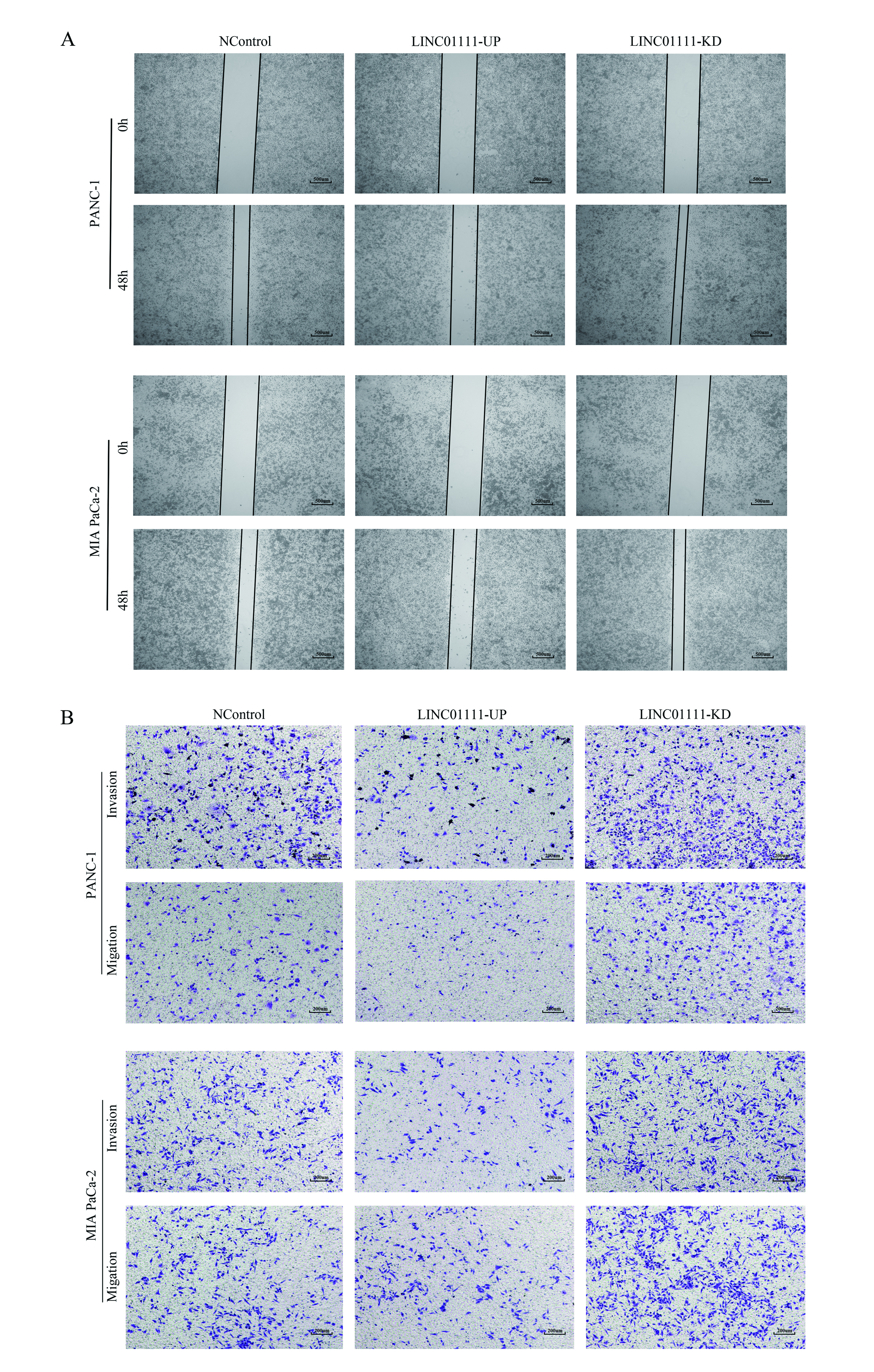

Supplement: Supplementary file 8 — Supplementary figure 3 [file 41419_2019_2123_MOESM8_ESM.tif]

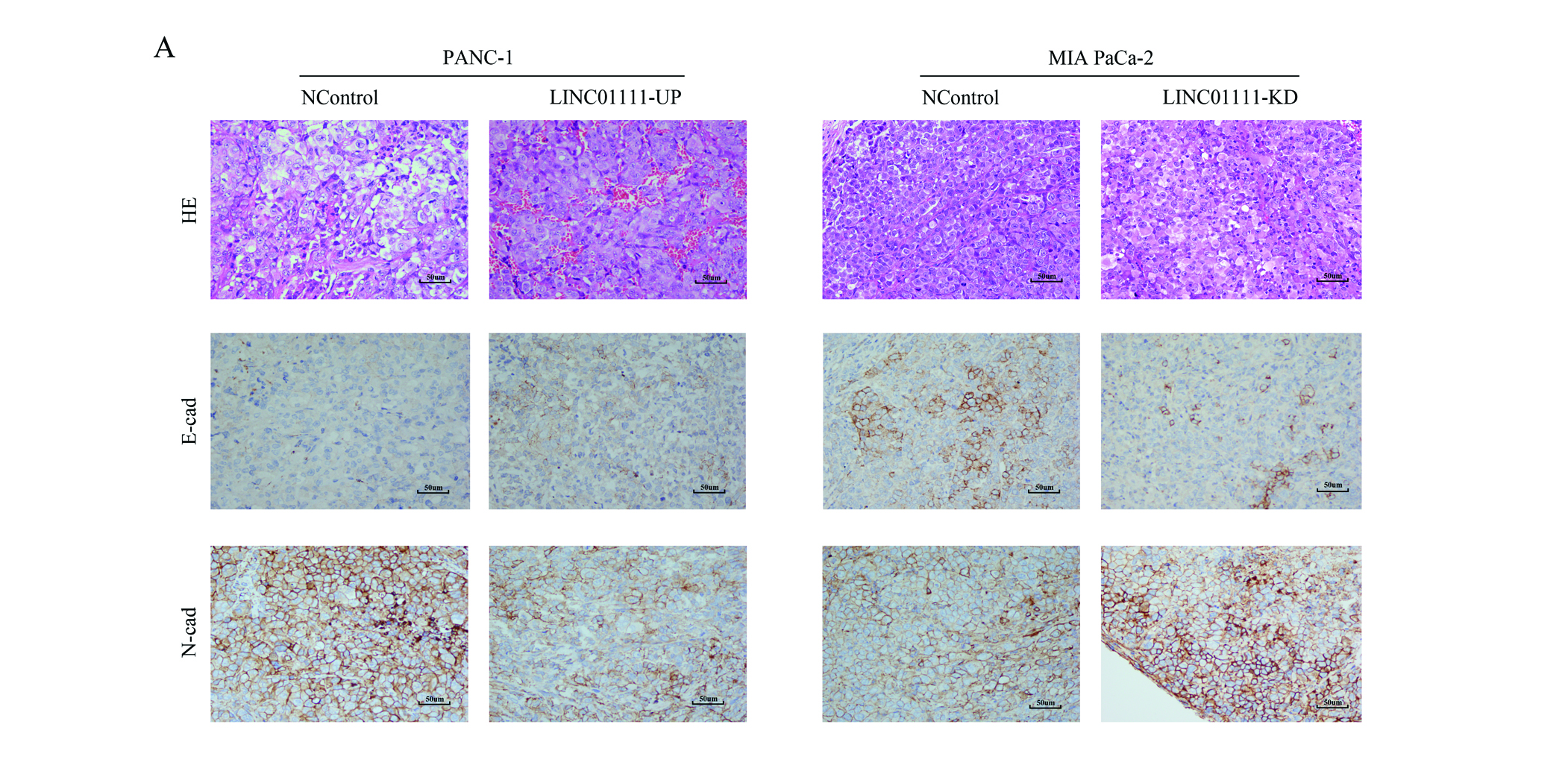

Supplement: Supplementary file 9 — Supplementary figure 4 [file 41419_2019_2123_MOESM9_ESM.tif]
